# Invasive Klebsiella Syndrome With Multiple Liver and Renal Abscesses

**DOI:** 10.7759/cureus.84052

**Published:** 2025-05-13

**Authors:** Pin Ru Tan, Natasha Mohd Nasran, Chee Yik Chang

**Affiliations:** 1 Medical Education, Newcastle University Medicine Malaysia (NUMed), Johor Bahru, MYS; 2 Radiology, MARA University of Technology, Selangor, MYS; 3 Infectious Diseases, Hospital Sultanah Aminah, Johor Bahru, MYS

**Keywords:** hypervirulent k. pneumoniae (hvkp), invasive klebsiella syndrome, klebsiella liver abscess, klebsiella pneumoniae (kp), multiple organ abscesses, perinephric collections, renal abscess

## Abstract

Invasive Klebsiella syndrome (IKS) is a significant health concern, particularly in Asia, as it results in a high mortality rate. Due to the hypervirulent strains of *Klebsiella pneumoniae*, it is more frequently detected among immunosuppressed individuals and will mainly metastasize to different organs, including the liver, kidneys, eyes, and brain. Here, we present the case of a 70-year-old man with underlying diabetes mellitus who developed multiple organ abscesses secondary to *K. pneumoniae*, requiring prolonged antibiotic treatment and abscess drainage. This case report highlights the importance of early diagnosis and appropriate antibiotic therapy to improve patient outcomes.

## Introduction

Invasive Klebsiella syndrome (IKS) is a severe and potentially life-threatening condition characterized by the spread of hypervirulent strains of *Klebsiella pneumoniae* to multiple organ systems [1,2]. This gram-negative bacterium, typically a part of the human gastrointestinal and respiratory microbiota, can cause serious infections in immunocompromised individuals, such as those with diabetes mellitus, chronic obstructive pulmonary disease (COPD), or those on immunosuppressive therapies [3]. 

*K. pneumoniae* is an opportunistic gram-negative bacterium known to cause a range of pyogenic infections, including liver abscesses, kidney abscesses, and endophthalmitis. A growing body of evidence links hypervirulent strains to more aggressive clinical outcomes [4,5]. The incidence of IKS has been notably rising, especially in East and Southeast Asia, due to the increasing prevalence of hypervirulent strains [6]. This case report highlights a 70-year-old male patient with underlying diabetes mellitus and multiple organ abscesses caused by a hypervirulent strain of *K. pneumoniae*. 

## Case presentation

A 70-year-old Chinese male patient with underlying diabetes mellitus, hypertension, and hyperlipidemia presented with a one-month history of intermittent fever accompanied by chills and rigors, reduced oral intake, generalized abdominal discomfort, and dysuria. His vital signs upon arrival at the emergency department were as follows: blood pressure of 121/65 mmHg, heart rate of 96 beats per minute, temperature of 39.1°C, respiratory rate of 18 breaths per minute, and oxygen saturation of 97% while breathing ambient air. He was alert and conscious. Auscultation of the lungs revealed bibasal crepitations. The abdomen was soft and non-tender, with no organomegaly. An eye examination revealed no abnormalities.

Full blood count showed leukocytosis with neutrophilia, with a white cell count of 38.2 × 10⁹/L (normal range: 4.0-10.0 × 10⁹/L) and a neutrophil count of 32.8 × 10⁹/L (normal range: 2.0-7.0 × 10⁹/L). Thrombocytosis was noted, with a platelet count of 933 × 10⁹/L (normal range: 150-410 × 10⁹/L). Normocytic normochromic anemia was present, with a hemoglobin level of 85 g/L (normal range: 130-170 g/L). The C-reactive protein (CRP) level was markedly elevated at 267 mg/L (normal range: <3 mg/L). His fasting glucose level was 6.5 mmol/L (normal range: 3.6-8 mol/L). A chest radiograph showed no abnormalities. The patient was initially treated for a suspected chest infection with intravenous amoxicillin-clavulanic acid (1.2 g every eight hours) and oral doxycycline (100 mg twice daily). The blood cultures subsequently identified *K. pneumoniae*, which was susceptible to amoxicillin-clavulanic acid, ampicillin-sulbactam, and ceftriaxone but resistant to ampicillin. Further analysis of the isolate revealed a hypervirulent strain of *K. pneumoniae*. The string test was positive, and the genes MagA, iroD, rmpA, and peg344 were detected.

**Figure 1:**
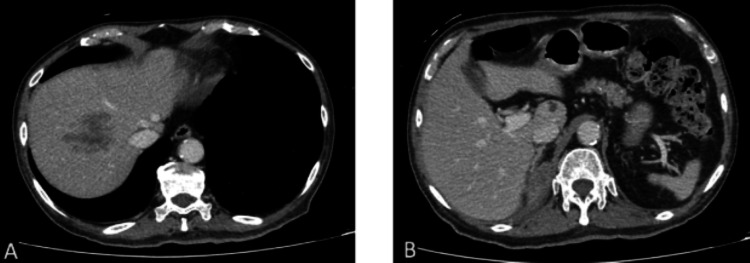
Axial CT images: (A) An ill-defined, heterogeneous hypodense lesion in segment VIII. (B) A smaller, ill-defined, heterogeneous hypodense lesion in segment I.

**Figure 2:**
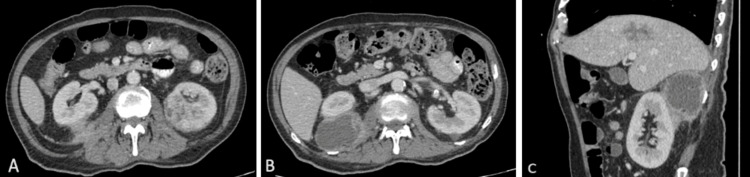
Axial and sagittal CT images: (A) Ill-defined hypodense lesions in both kidneys; (B-C) a large, rim-enhancing right perinephric collection with areas of liquefaction.

A contrast-enhanced computed tomography (CT) scan of the abdomen and pelvis showed ill-defined heterogeneous hypodense lesions at segment VIII of the liver, suggesting non-liquefied liver abscess (Figure 1A), measuring 4.2 × 6.0 × 3.3 cm (AP x W x CC). Another small lesion of similar appearance is seen at the caudate lobe (Figure 1B), measuring 1.0 × 1.0 × 1.2 cm. Additionally, there were also multiple other abscesses in both kidneys (Figure 2A), with a large perinephric collection seen on the right (Figures 2B-2C), measuring 5.3 × 7.0 × 9.5 cm. An ultrasound-guided pigtail drainage of the right perinephric collection was performed, draining 50 mL of foul-smelling hemopurulent fluid. The fluid culture yielded *K. pneumoniae* with an identical susceptibility profile as the blood culture. The pigtail catheter was removed after two weeks, as the drain output was minimal. 

Following two weeks of antibiotic therapy, significant clinical improvement was observed. Repeated imaging demonstrated a reduction in the size of the liver and renal abscesses, as well as the right perinephric collection. A follow-up imaging study performed one month (i.e., 4 weeks) after the commencement of antibiotics showed resolution of the bilateral renal abscesses and right perinephric collection, with significant improvement of the liver abscesses and a small residual lesion remaining in segment VIII.

Due to the clinical and radiological improvements, the patient was discharged home with oral ampicillin-sulbactam for another two weeks. Follow-up imaging at six weeks of antibiotic therapy showed complete resolution of the residual liver abscess, with no new focal abscesses seen. The antibiotic therapy was discontinued, and the patient was scheduled for an outpatient review at the infectious diseases clinic.

## Discussion

IKS is a potentially life-threatening condition caused by hypervirulent strains of* K. pneumoniae* [1,2]. This gram-negative facultative anaerobic bacillus is a common colonizer of human mucosal surfaces, particularly the nasopharynx and gastrointestinal tract. However, in immunocompromised individuals such as those with diabetes mellitus, COPD, organ transplantation, or prolonged glucocorticoid therapy, *K. pneumoniae* can invade multiple organ systems, leading to severe disseminated infections [3].

Due to its extensive presence in the human microbiota, *K. pneumoniae *from a primary source of infection can spread hematogenously to distant sites, including the liver, kidneys, prostate, brain, and eyes. As a result, it is a major causative agent of pyogenic liver abscess, meningitis, pneumonia, and endophthalmitis [1-3]. A particularly rare manifestation is *K. pneumoniae*-associated prostate abscess, which has been reported in previous cases, demonstrating the diverse clinical presentations of IKS [4].

Over the past three decades, the incidence of *K. pneumoniae* pyogenic liver abscess has risen significantly in East Asian countries, particularly Taiwan, likely due to the increasing prevalence of hypervirulent strains. A retrospective analysis in New York revealed that 78.3% of patients diagnosed with *K. pneumoniae* liver abscess had origins in Asia, highlighting a strong epidemiological link [5]. Additionally, a study by Lin et al. analyzing 954 stool specimens from Chinese adults across multiple Asian countries found that 62.1% carried *K. pneumoniae*, with Malaysia exhibiting the highest colonization rate (87.7%), followed by Taiwan (75%), Singapore (61.1%), and Japan (18.8%). These findings suggest a significant burden of *K. pneumoniae* colonization in Southeast Asia, increasing the risk of invasive disease in susceptible populations [6].

Given the aggressive nature of IKS, a high degree of clinical suspicion is essential, particularly in patients with *K. pneumoniae *bacteremia. Early diagnosis is crucial to prevent complications, and imaging plays a key role in identifying metastatic infections [7]. Abdominal ultrasonography or contrast-enhanced CT scans are recommended for detecting liver and prostate abscesses, while MRI brain and fundoscopy are necessary for evaluating possible meningitis and endophthalmitis [8]. 

The mainstay of treatment involves prompt initiation of antibiotic therapy guided by susceptibility testing, as hypervirulent *K. pneumoniae* strains may exhibit antimicrobial resistance. Empirical treatment typically includes third-generation cephalosporins or carbapenems in cases of multidrug resistance [9]. Additionally, abscess drainage is often required for source control, particularly in cases of pyogenic liver or prostate abscesses. Close monitoring and early intervention are essential to improve patient outcomes and reduce mortality associated with IKS [10].

## Conclusions

By reviewing the prevalence of IKS in East and Southeast Asian countries, it is worthwhile to consider *K. pneumoniae *as an etiological factor, especially in patients with uncontrolled diabetes mellitus developing clinical sepsis. This case report highlights the importance of early clinical suspicion and timely intervention to minimize complications. Further research is required to gain a deeper insight into the pathogenesis of IKS and to provide more effective therapeutic approaches.
